# A 40-year-old female with III-degree utero cervical descent

**DOI:** 10.11604/pamj.2023.45.128.40650

**Published:** 2023-07-17

**Authors:** Dharti Khewale, Manjusha Mahakalkar

**Affiliations:** 1 Department of Obstetrics and Gynaecology Nursing, Srimati Radhikabai Meghe Memorial College of Nursing, Datta Meghe Institue of Higher Education and Research (DU) Sawangi Meghe, Maharashtra, Wardha, India

**Keywords:** Uterine prolapse, cervicitis, cervical descent, pelvic organ prolapse

## Image in medicine

Inside the pelvis, the uterus (or womb) is normally situated with various muscles, tissue, and ligaments. These muscles get weak after pregnancy, childbirth and difficult labor in women. A prolapsed uterus is a disorder that occurs when a woman's uterus drops into the vaginal canal as she ages or due to a natural depletion of the hormone oestrogen. We report the case of a 40-year-old female who was brought by her son to the hospital. The patient reported that something coming out of the vagina for 2 years, and it was progressive in nature. The cervical biopsy was done, and the histopathology report showed chronic cervicitis with hyperkeratosis and the ultrasonography showed severe cystitis and bilateral hydronephrosis. The vaginal examination revealed the patient had 3^rd^ degree utero cervical descent. A hysterectomy was done, and postoperatively she was treated with intravenous analgesics, and antibiotics during hospitalization.

**Figure 1:**
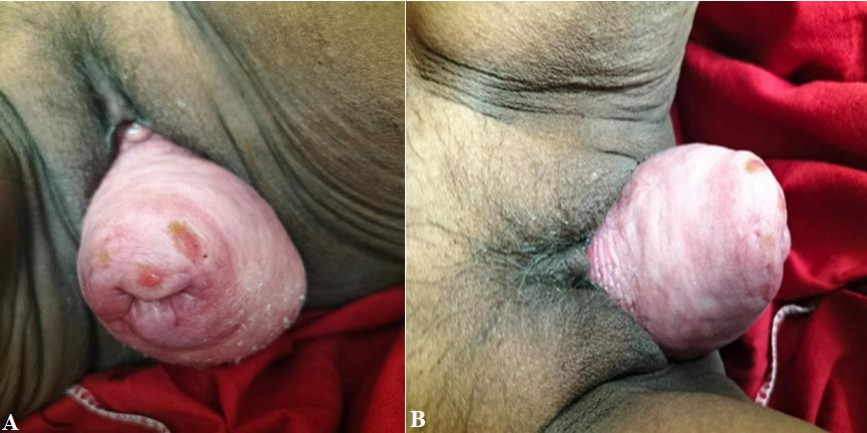
A,B) III degree utero cervical descent

